# Supplementary material for: Size-dependent stability of ultra-small α-/β-phase tin nanocrystals synthesized by microplasma
Source: Nat Commun. 2019 Feb 18;10:817. doi: 10.1038/s41467-019-08661-9 (PMC6379433; doi:10.1038/s41467-019-08661-9)
Supplement: Supplementary file 1 — Supplementary Information [file 41467_2019_8661_MOESM1_ESM.pdf]

Supplementary Information

Size-dependent stability of ultra-small α-/β- phase tin nanocrystals synthesized by microplasma

Haq et al.

Supplementary note

Bohr exciton radius of semiconducting tin (i.e. α-Sn) is calculated by using the following Equation (1) as follows;[1]

$$a_B = \frac{m_o * \varepsilon_r * r_{B,H}}{\mu} \qquad (1)$$

where m_o is the electron mass, ε_r is the relative dielectric permittivity, $r_{B,H}$ is the Bohr radius of hydrogen and μ is the reduced effective electron-hole mass. The ε_r for α-Sn has been reported as 24 nm at 300 K.[2]

Supplementary Table 1: The Bohr exciton radius estimation for α-Sn that resulted in an exciton radius of around 12.6 nm.

effective mass of electron (m_e)	effective mass of hole (m_h)	Reduced effective mass (μ)	Bohr radius of hydrogen $r_{B,H}$ / nm	Bohr radius of α-Sn a_B / nm
**0.21m_o** [2]	0.195m_o [2]	0.101 [2]	0.053 [1]	**12.56**

Dependence of the NCs diameter on the gas flow rate

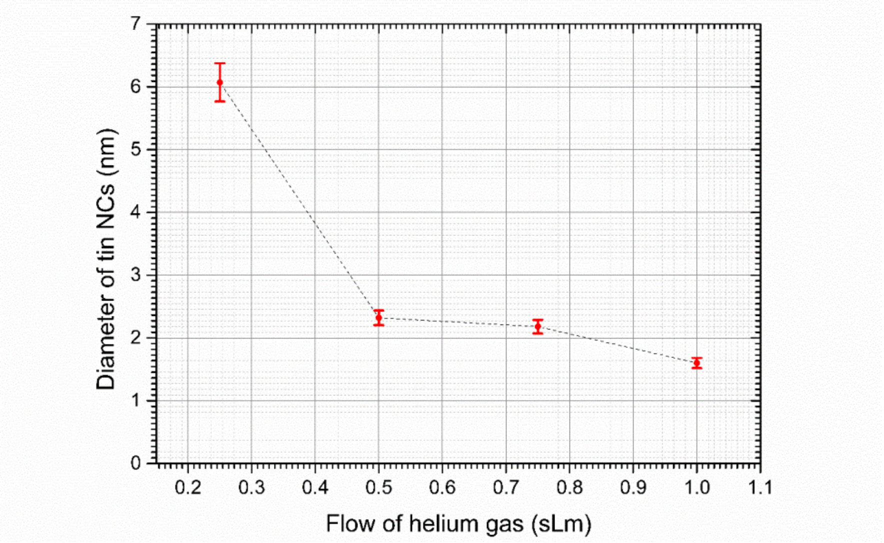

Supplementary Figure 1: Diameter of the tin (Sn) nanocrystals (NCs) as a function of gas flow rates of helium in our experimental plasma setup.

Transmission electron microscopy analysis

Supplementary Table 2: Summary of lattice spacing expected and measured by high resolution transmission electron microscopy (HRTEM) and selective area electron diffraction (SAED) for the different sized NCs. The expected interplanar spacing (nm) is taken from the standard crystallographic databases i.e. JCPDS: 01-089-4898 for β-Sn and JCPDS: 01-089-4789 for α-Sn.

Phase	Plane	Expected Spacing (nm)	6.07 nm NCs Measured Spacing (nm)	2.32 nm NCs Measured Spacing (nm)	2.18 nm NCs Measured Spacing (nm)	1.60 nm NCs Measured Spacing (nm)
β-Sn	(101)	0.279	0.263 (SAED)	-	0.277 (SAED)	-
β-Sn	(112)	0.148	-	-	0.150 (SAED)	-
β-Sn	(200)	0.291	-	0.290 (HRTEM)	-	-
β-Sn	(211)	0.201	0.200 (HRTEM)<br>0.202 (SAED)	0.203 (SAED)	-	-
β-Sn	(220)	0.206	0.212 (SAED)	-	-	-
β-Sn	(301)	0.165	0.160 (HRTEM)<br>0.175 (SAED)	0.161 (SAED)	0.160 (HRTEM)<br>0.166 (SAED)	-
β-Sn	(321)	0.144	0.135 (SAED)	0.142 (SAED)	-	-
α-Sn	(111)	0.374			0.365 (SAED)	-
α-Sn	(220)	0.229	-	0.233 (SAED)	0.225 (SAED)	0.223 (SAED)
α-Sn	(222)	0.187	-	0.180 (HRTEM)	0.180 (HRTEM)	0.180 (HRTEM)
α-Sn	(311)	0.195	-	-	0.190 (HRTEM)<br>0.196 (SAED)	0.190 (HRTEM)<br>0.197 (SAED)
α-Sn	(331)	0.148	-	-	-	0.139 (SAED)
α-Sn	(511)	0.124	-	-	-	0.115 (SAED)

The analysis shows that differences between expected and measured values are all below 7.5%. This is consistent with experimental errors and deviation due to nanoscale dimensions.[3] Furthere below we report here also representative HR-TEM images with corresponding evaluation of the lattice fringes and fast Fourier Transforms.

Additional HR-TEM images and corresponding FFT for 6.1 nm

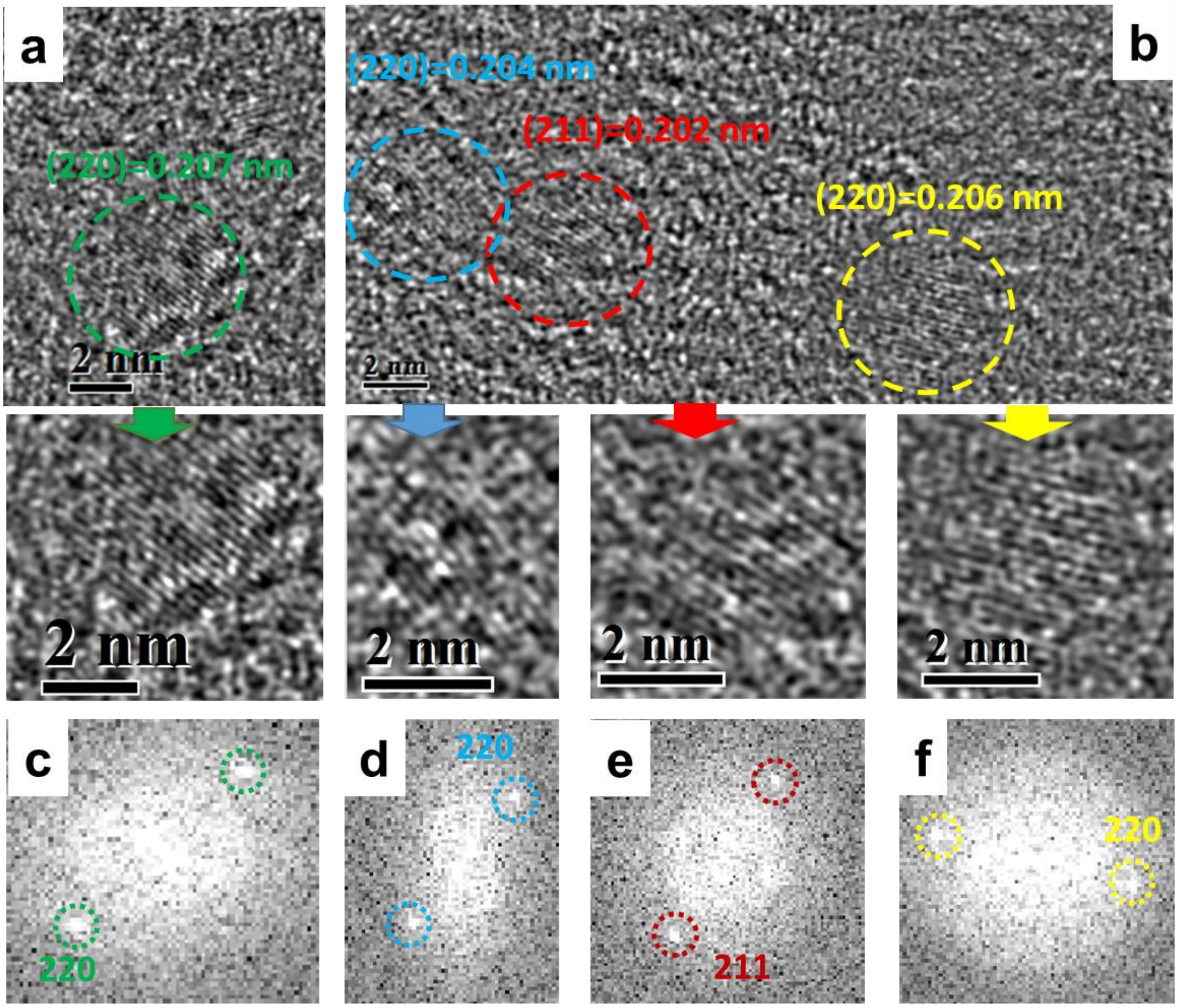

Supplementary Figure 2: Structural characterization of tin nanocrystals. (a-b) High-resolution transmission electron microscopy (HR-TEM) images of four β-Sn nanocrystals (NCs) showing their lattice fringes and their lattice spacings; **(c-f)** the fast Fourier transforms (FFTs) images of the corresponding HR-TEM images.

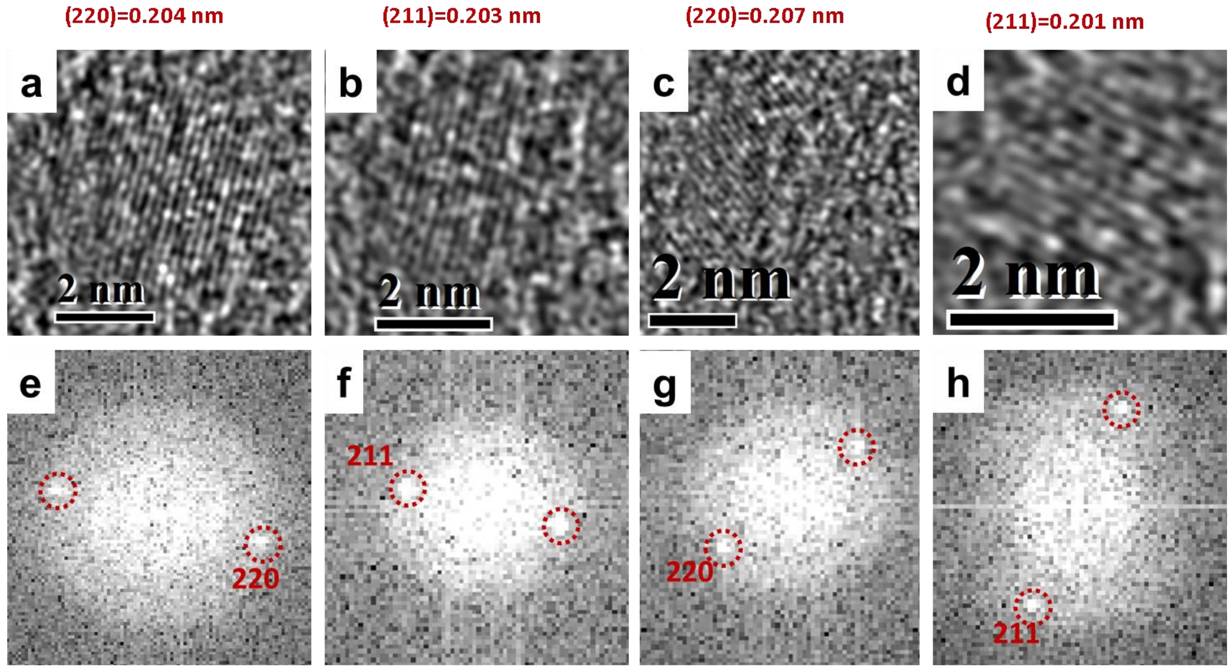

Supplementary Figure 3: Structural characterization of tin nanocrystals. (a-d) High-resolution transmission electron microscopy (HR-TEM) images of β-Sn nanocrystals; **(e-h)** fast Fourier transforms (FFTs) images of the corresponding HR-TEM images.

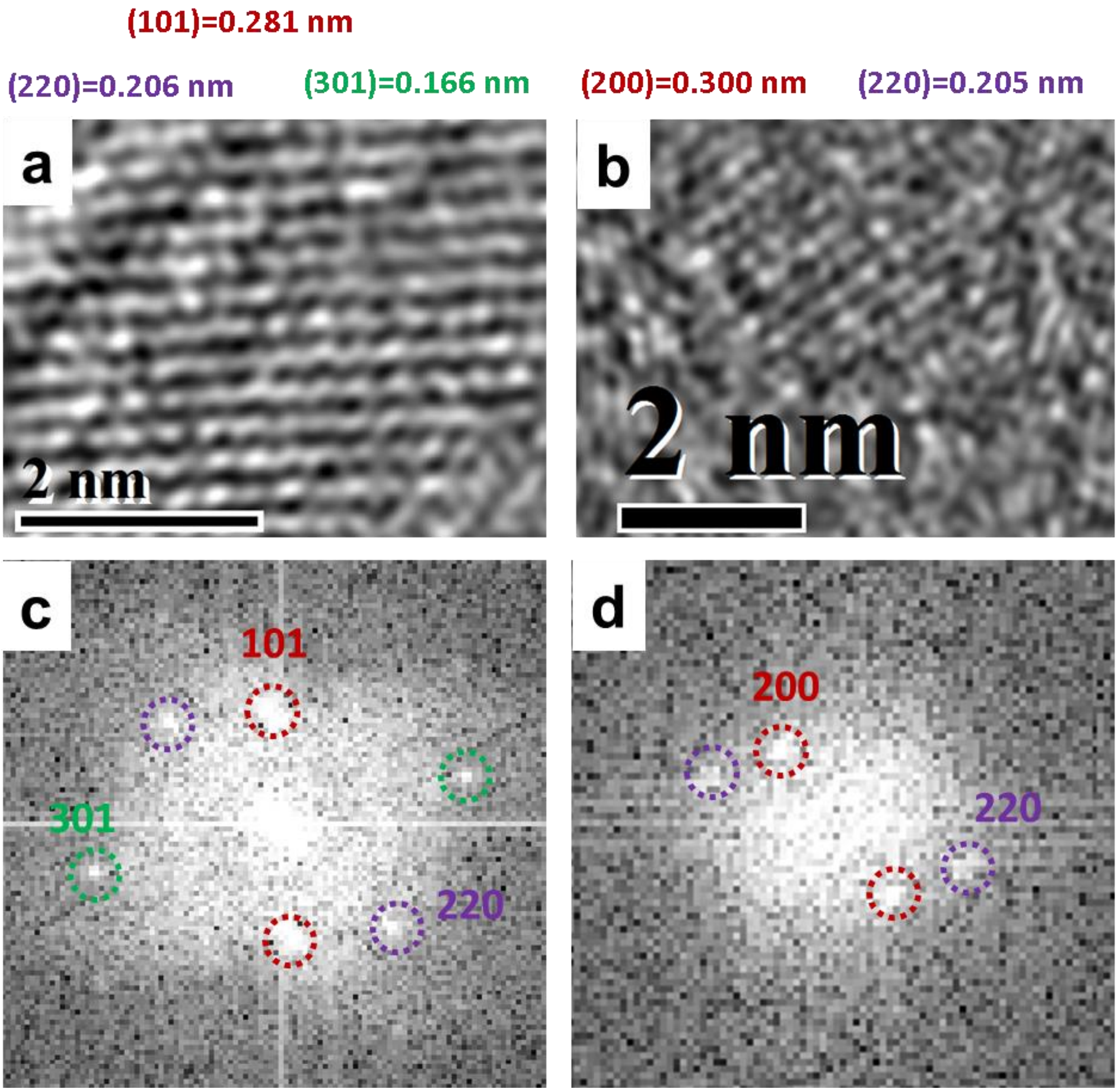

Supplementary Figure 4: Structural characterization of tin nanocrystals. (a-b) High-resolution transmission electron microscopy (HR-TEM) images of β-Sn nanocrystals; **(c-d)** fast Fourier transforms (FFTs) images of the corresponding HR-TEM images respectively.

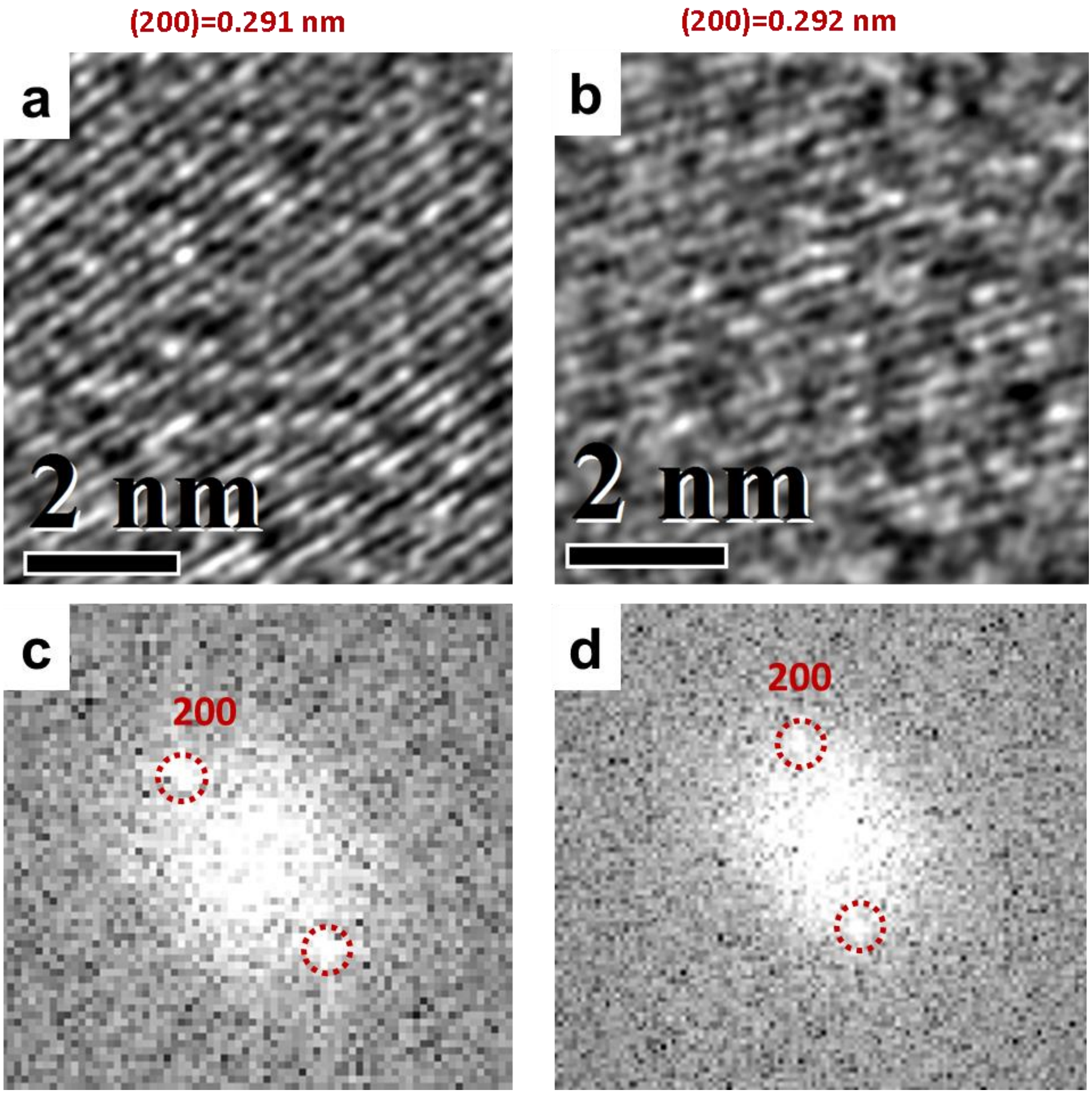

Supplementary Figure 5: Structural characterization of tin nanocrystals. (a-b) High-resolution transmission electron microscopy (HR-TEM) images of β-Sn nanocrystals; **(c-d)** fast Fourier transforms (FFTs) images of the corresponding HR-TEM images respectively.

Additional HR-TEM images and corresponding FFT for 1.6 nm Sn NCs

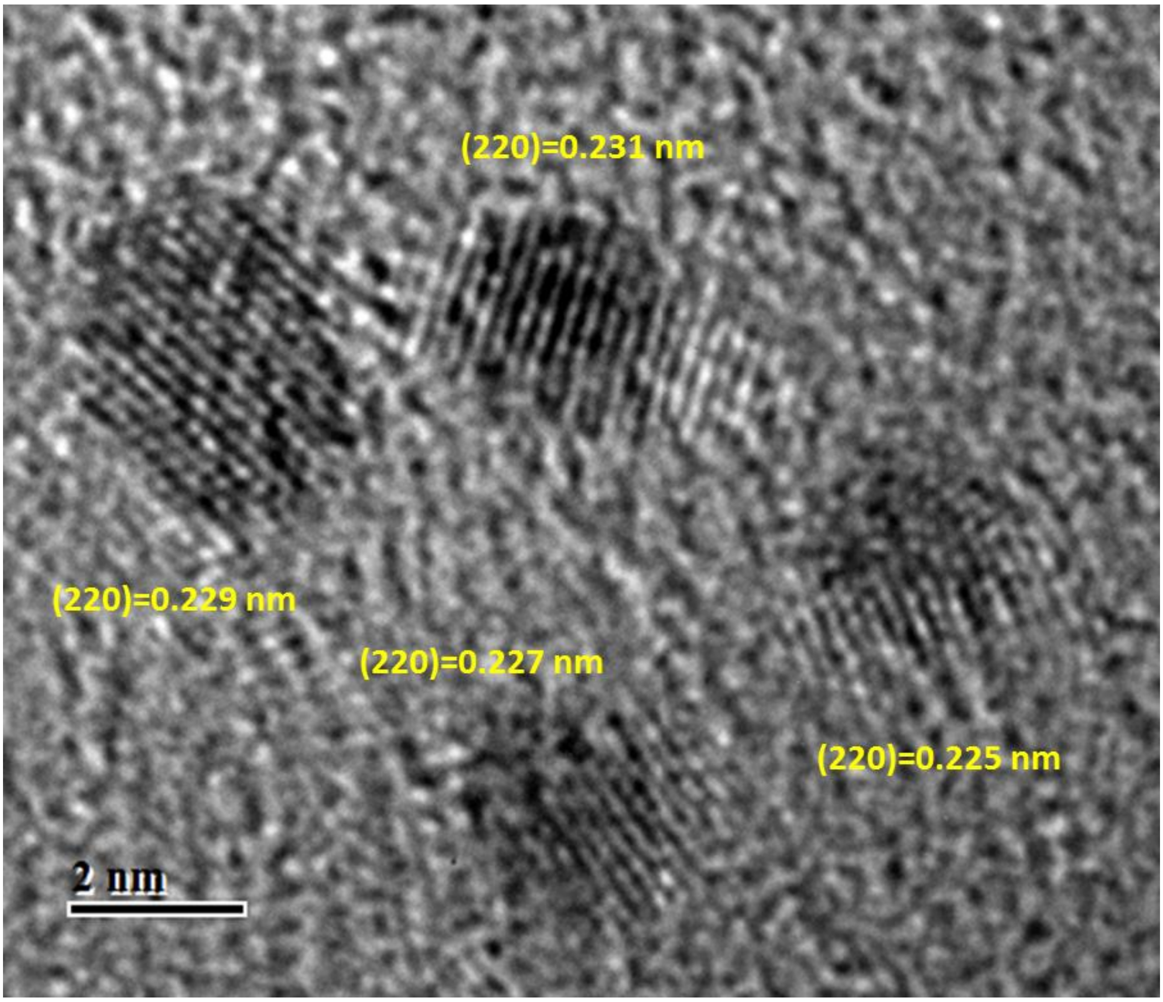

Supplementary Figure 6: Structural characterization of tin nanocrystals. High-resolution transmission electron microscopy (HR-TEM) image of α-Sn nanocrystals showing the lattice fringes and their spacings corresponding to (220) planes.

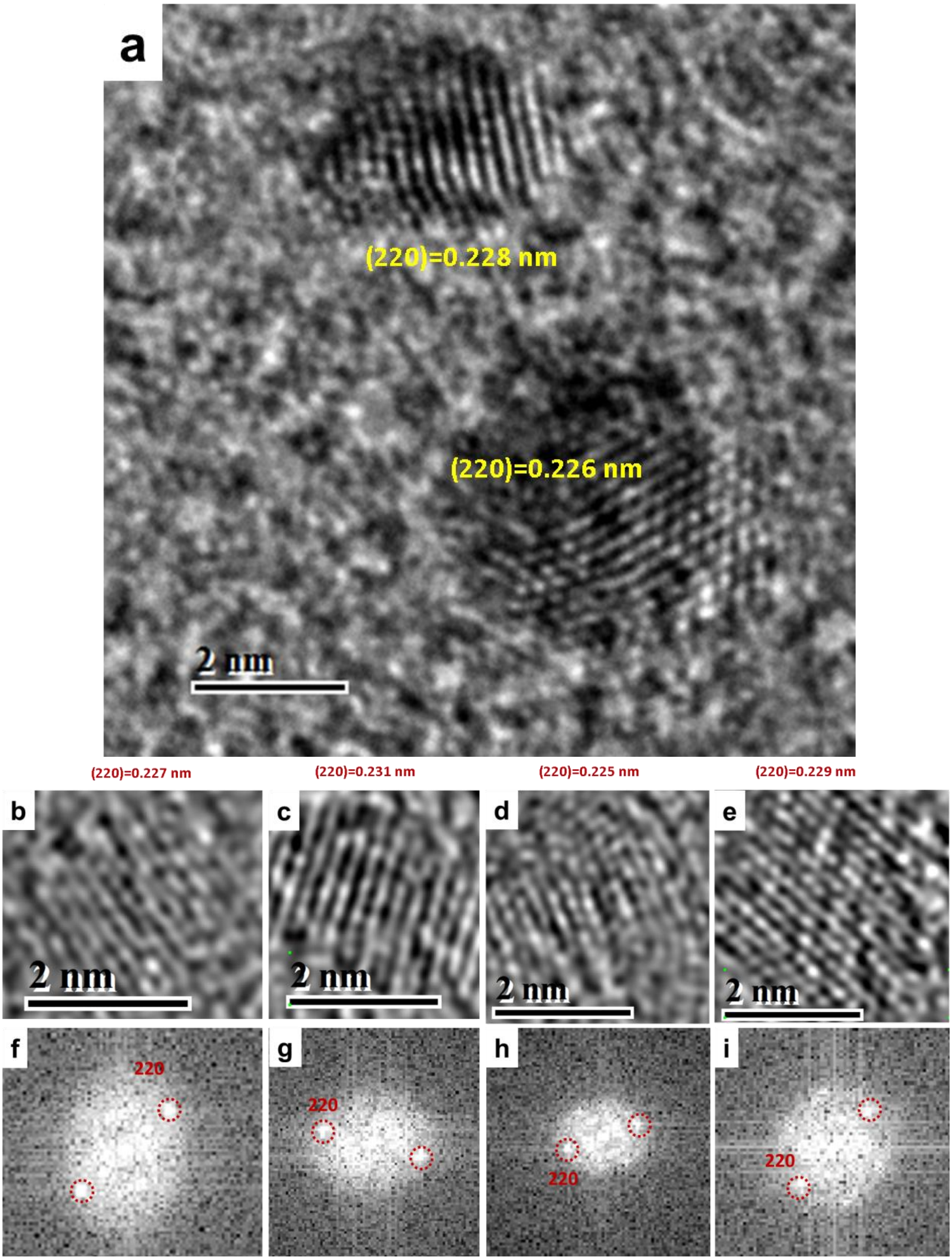

Supplementary Figure 7: Structural characterization of tin nanocrystals. (a) High-resolution transmission electron microscopy (HR-TEM) images of α-Sn nanocrystals showing the lattice fringes spacings and the corresponding atomic planes; **(b-e)** HR-TEM images α-Sn NCs; **(f-i)** fast Fourier transforms (FFTs) images of the corresponding HR-TEM images (b-e).

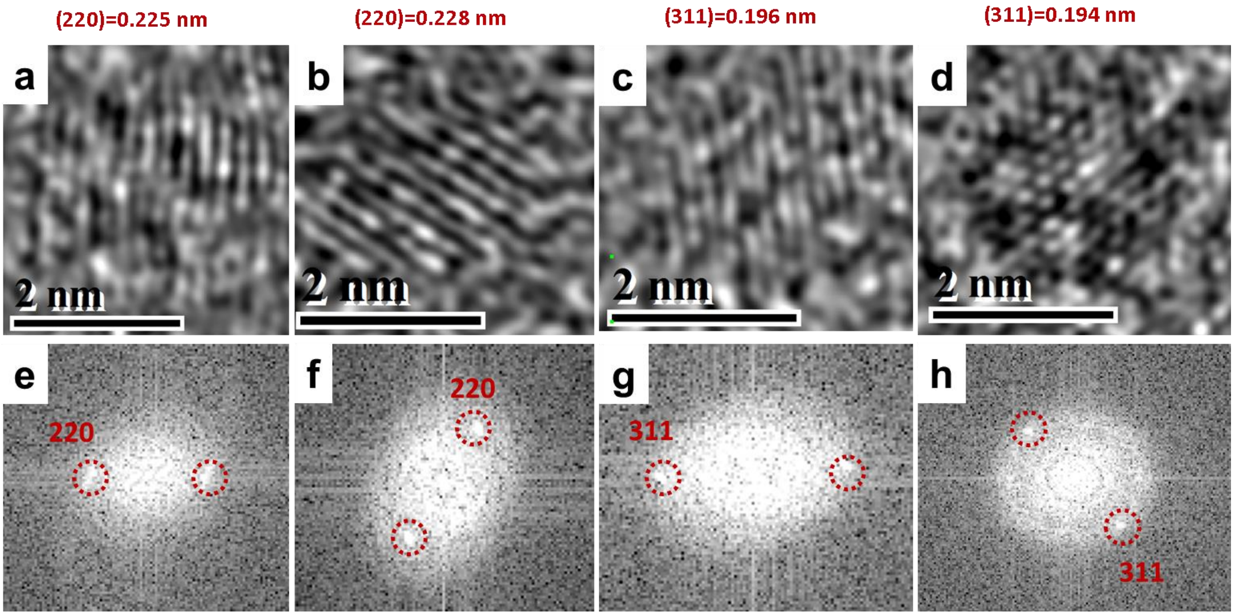

Supplementary Figure 8: Structural characterization of tin nanocrystals. (a-d) High-resolution transmission electron microscopy (HR-TEM) images of α-Sn nanocrystals showing the lattice fringes spacings and the corresponding planes; **(e-h)** fast Fourier transforms (FFTs) images of the corresponding HR-TEM images (a-d).

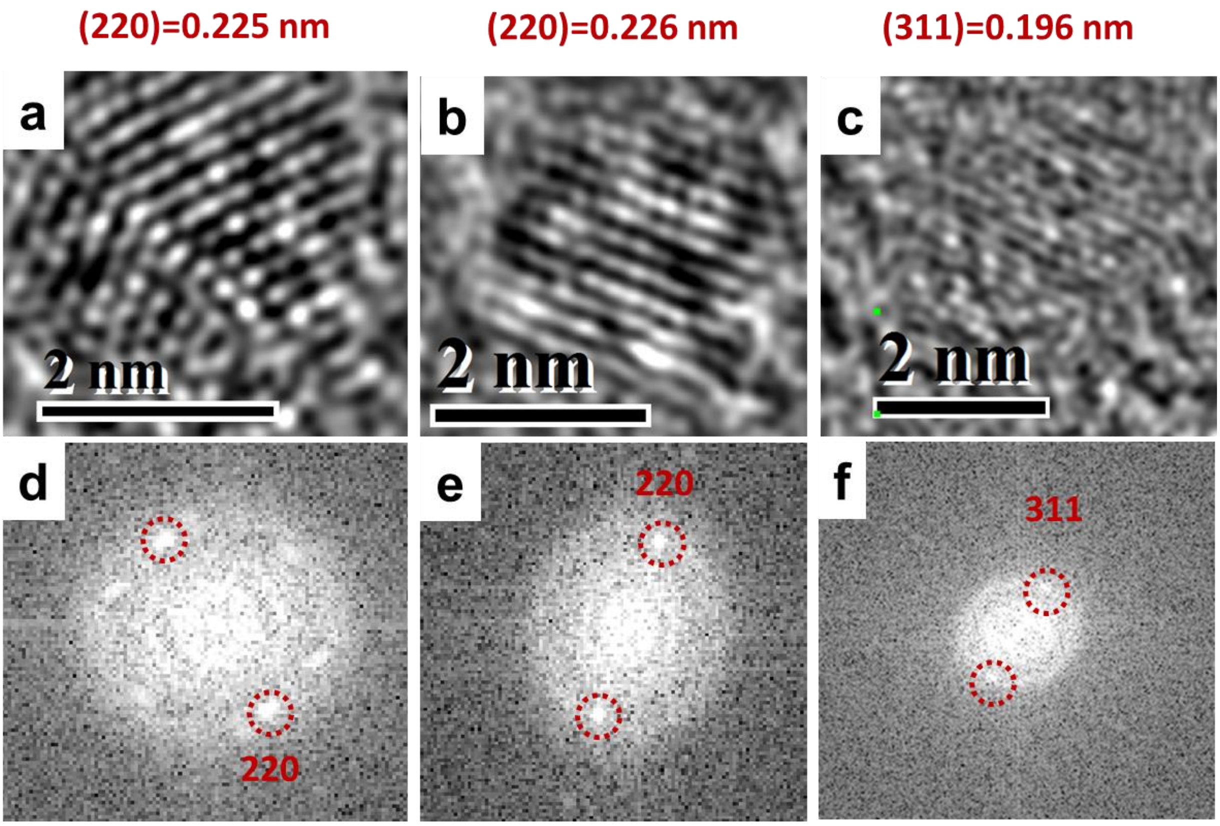

Supplementary Figure 9: Structural characterization of tin nanocrystals. (a-c) High-resolution transmission electron microscopy (HR-TEM) images of α-Sn nanocrystals showing the lattice fringes spacings and the corresponding atomic planes; **(d-f)** fast Fourier transforms (FFTs) images of the corresponding HR-TEM images in (a-c).

Transmittance measurements for 1.6 nm Sn NCs

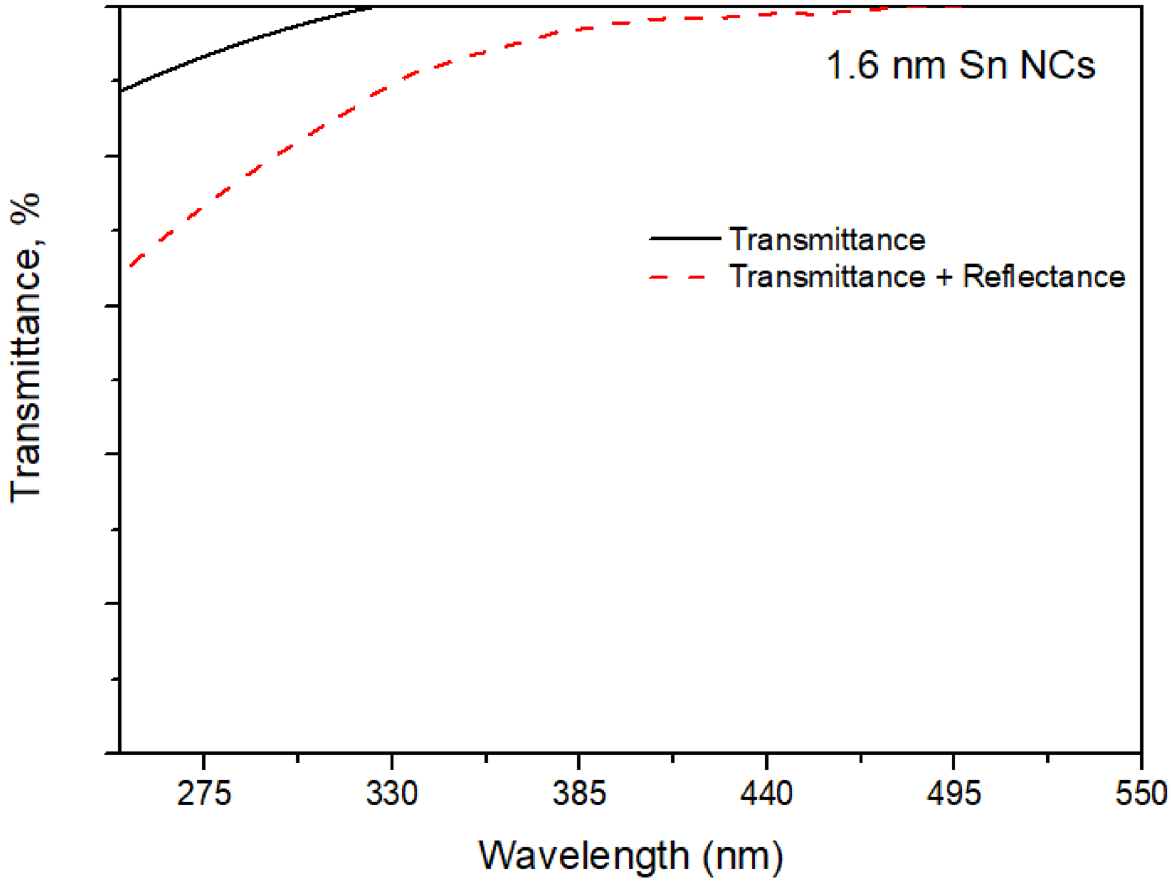

Supplementary Figure 10: UV-Vis measurements carried out in standard transmission mode (black line) and inside the integrating sphere (red dashed line).

The bandgap is calculated using the following Tauc plot as reported in the main manuscript. In order to determine the relative absorption coefficient as needed for the Tauc plot, the following relations has been used:

$$C\alpha = \frac{1-(T+R)}{1-T}(-\ln(T)) \quad (2)$$

$$\alpha h\nu = C'(h\nu - E_g)^n \quad (3)$$

Where α is the absorption coefficient, *C* and *C'* are proportionality constants, $h\nu$ is the photon energy, *T* and *R* are transmittance and reflectance respectively, E_g is the bandgap and *n* is the optical transitions. The value of *n* is taken as 1/2 for direct bandgap transitions.

Supplementary note

The electrochemical performance of Sn NCs were evaluated from cyclic voltammograms obtained using the three electrode electrochemical setup as shown in Supplementary Figure 11. The areal capacitance of the sample was estimated by using the following relation:

$$C = \frac{\int IdV}{As\Delta V} . \qquad (4)$$

where, *A* is the area of the working electrode (WE), *s* is the scan rate, $\int IdV$ is the integral area of the cyclic voltammogram and ΔV stands for the potential window.

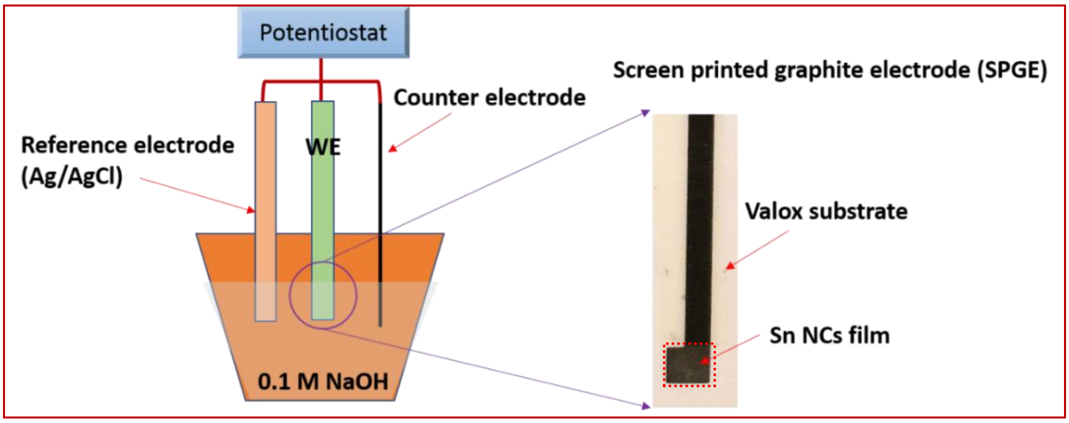

Supplementary Figure 11: Electrochemical setup. A schematic diagram showing the three-electrode electrochemical setup for performing cyclic voltammetry of Sn NCs in 0.1 M NaOH solution. A digital photograph of the working electrode (WE) with active geometric area of 0.04 cm^2 (red dotted square) is also shown in the figure. Only the active geometric area containing Sn NCs was exposed to the electrolyte.

Screen printed electrodes (SPE) were fabricated using a DEK 240 Manual Screen Printing Machine, a stainless-steel screen mesh and graphite ink (Gwent Electronic Materials (GEM) Product code: C205010697)[4]. The SPE were screen printed in groups of eight onto a Valox substrate (purchased from Cardillac Plastics, UK) which was cured at 70 °C for 90 min[4]. A dielectric material was used to define the working area of the electrode which in our case is 0.04 cm^2 as also shown in Supplementary Figure 11.

Here below (Supplementary Figure 12) we include the electrochemical performance for all NCs including those which exhibited mixed phases. Structurally mixed phases in Sn NCs

(with diameter of 2.18 nm and 2.32 nm produced with flow rates at 0.50 sLm and 0.75 sLm) demonstrated lower and unstable performance, possibly driven by unstable mixed phases and increased internal resistance offered by larger NCs. Furthermore for these NCs, the degree of one phase vs. the other was difficult to estimate and control, hence it was difficult to clearly assess their preformance in relation to their materials properties. For these reasons we have not included these in the discussion in the main manuscript.

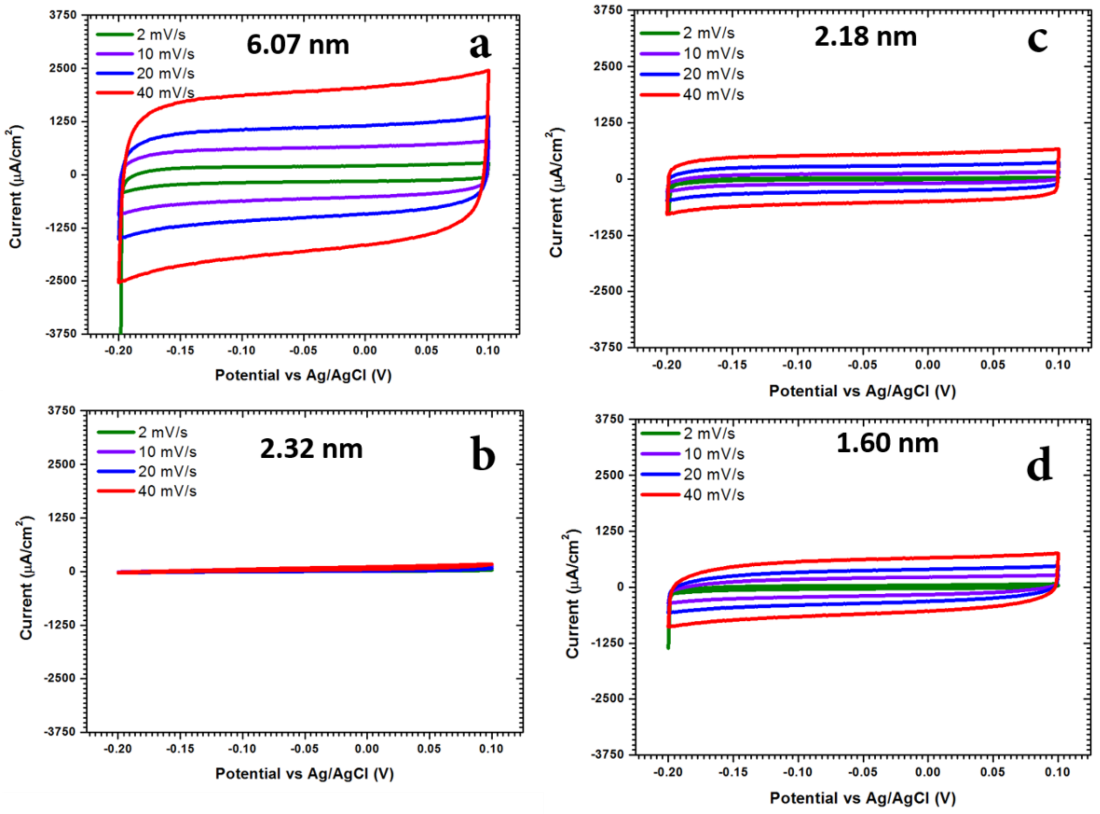

Supplementary Figure 12: **Electrochemical performance of tin nanocrystals.** Cyclic voltammograms of **(a)** 6.07 nm, **(b)** 2.32 nm, **(c)** 2.18 nm and **(d)** 1.60 nm tin nanocrystals attained at the scan rates of 2 mV s^{-1}, 10 mV s^{-1}, 20 mV s^{-1} and 40 mV s^{-1} respectively.

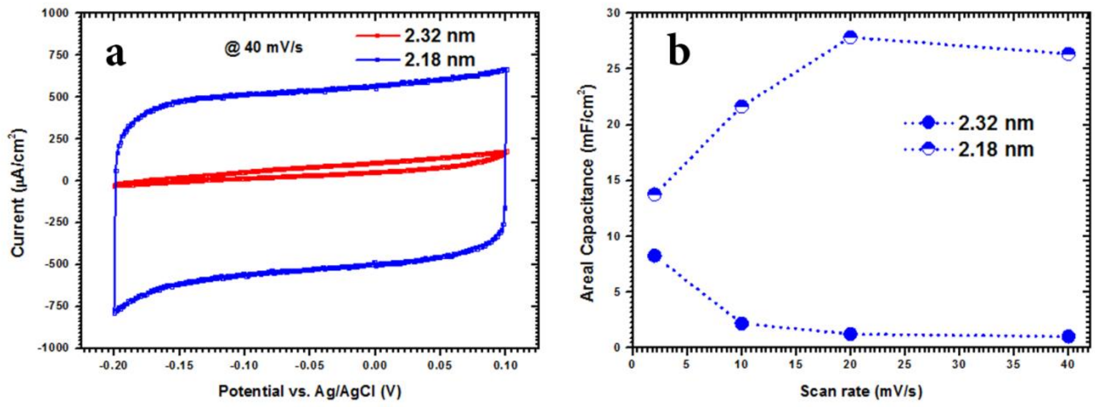

Supplementary Figure 13: Electrochemical performance of tin nanocrystals. (a) Cyclic voltammograms of 2.18 nm (at 0.75 sLm) and 2.32 nm (at 0.5 sLm) tin (Sn) nanocrystals (NCs) in 0.1 M sodium hydroxide (NaOH) solution attained at a scan rate of 40 mV s^{-1}; **(b)** Evolution of the relative areal capacitances of 2.18 nm and 2.32 nm Sn NCs versus the scan rates of 2 mV s^{-1}, 10 mV s^{-1}, 20 mV s^{-1} and 40 mV s^{-1}.

The cyclic voltammograms (at 40 mVs^{-1}) and the variations in areal capacitances for the 2.18 nm and 2.23 nm Sn NCs (containing mixed-phases) are shown in Supplementary Figure 13a and Supplementary Figure 13b respectively. Generally, the capacitance decreases with an increase in scan rate due to insufficient time for the electrolyte to adsorb and desorb on the electrode surface. In our case, the areal capacitance of 2.32 nm Sn NCs decreased initially with increasing scan rate, whereas the 2.18 nm Sn NCs followed an opposite trend. This unexpected behavior for the 2.18 nm Sn NCs most probably arises from the presence if mixed phases, which lead to poor interfacial adhesion. In this way, the poor packing of mixed phases leads to increase in surface intercalation (or adsorption/desorption) of ions even at higher scan rates. Similarly, such interesting and unusual behavior in the evolution of capacitance over scan rate has also been noticed with other materials like nanoporous metal/oxides, where the authors have attributed the rise in capacitance with scan rate to the nanoporous nature of electrodes, which allows easy diffusion of ions at high scan rates.[5] Another reason could be attributed to the relatively lower resistance to ions surface intercalation in the cubic structure compared to tetragonal one.

Supplementary Figure 14a shows the CV of bare carbon electrodes in 0.1 M NaOH solution at a scan rate of 40 mV/s. Supplementary Figure 14b represents the variation of arial capacitance of bare carbon with increasing scan rate from 2 mV/s to 40 mV/s. The capacitance decreases with increasing the scan rate and the capacitance values are much lower than Sn NCs as discussed in main manuscript.

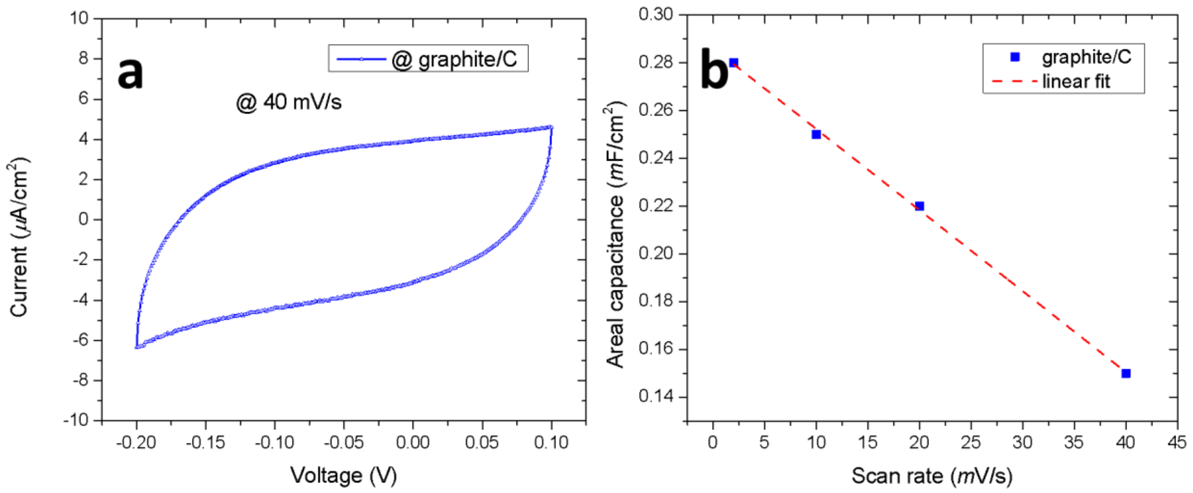

Supplementary Figure 14: Electrochemical performance of carbon electrodes. (a) Cyclic voltammogram bare carbon electrodes in 0.1 M sodium hydroxide (NaOH) solution attained at a scan rate of 40 mV s^{-1}; **(b)** Evolution of the areal capacitances of bare carbon versus the scan rates of 2 mV s^{-1}, 10 mV s^{-1}, 20 mV s^{-1} and 40 mV s^{-1}.

Supplementary note

A high precision microbalance (RADWAG, model MYA 2.4Y) was used to estimate the mass of Sn NCs deposited (for 30 min) on carbon electrodes. The mass of carbon electrodes before and after Sn deposition was measured. Hence, the difference in mass of carbon electrodes before and after the deposition of Sn NCs were calculated which estimates to the mass of Sn NCs. The production rates estimated in this way are reported in the Supplementary Table 3.

Supplementary Table 3: Summary of the deposition rates obtained with our microplasma set-up for 1.6 nm and 6.1 nm tin nanocrystals.

NC diameter	Deposition rate / µg min^{-1}
1.6 nm	4.7
1.6 nm	6.0
6.1 nm	2.2
6.1 nm	2.6

References

1. Shaalan, N. M., Hamad, D., Abdel-Latief, A. Y. & Abdel-Rahim, M. A. Preparation of quantum size of tin oxide: Structural and physical characterization. *Prog. Nat. Sci. Mater. Int.* **26,** 145–151 (2016).
2. Berger, L. I. *Semiconductor Materials*. (Taylor & Francis, 1996).
3. Ragan, R. & Atwater, H. A. Diamond cubic Sn-rich nanocrystals: Synthesis, microstructure and optical properties. *Appl. Phys. A Mater. Sci. Process.* **80,** 1335–1338 (2005).
4. Kanyong, P., Rawlinson, S. & Davis, J. Simultaneous electrochemical determination of dopamine and 5-hydroxyindoleacetic acid in urine using a screen-printed graphite electrode modified with gold nanoparticles. *Anal. Bioanal. Chem.* 1–9 (2016). doi:10.1007/s00216-016-9351-0
5. Lang, X., Hirata, A., Fujita, T. & Chen, M. Nanoporous metal/oxide hybrid electrodes for electrochemical supercapacitors. *Nat. Nanotechnol.* **6,** 232–236 (2011).
